# P04.02. How familiar are infectious disease (ID) physicians with integrative medicine (IM) modalities and are they willing to recommend them?

**DOI:** 10.1186/1472-6882-12-S1-P272

**Published:** 2012-06-12

**Authors:** K Shere-Wolfe, J Tilburt, C D'Adamo, B Berman, M Chesney

**Affiliations:** 1 University of Maryland, Baltimore, USA; 2 Mayo Clinic, Rochester, USA; 3 University of California, San Francisco, San Francisco, USA

## Purpose

The purpose of this study was to assess ID physicians’ familiarity and use of various IM modalities.

## Methods

A national survey of 1,000 practicing ID physicians in the United States was conducted in 2010. The IM modalities studied were mind-body based modalities (MBM), biologically based modalities (BBM), manipulative and body-based modalities (MBBM), energy based modalities (EBM) and whole medical systems (WMS). Participants were asked to indicate their familiarity of each modality. If they were familiar with the modality they were then asked to indicate whether they recommended it to patients.

## Results

A total of 311 (31%) ID physicians responded to the survey. The mean age was 49 years and 64% were male. ID physicians were most familiar with the following modalities (n=311)*: vitamins and mineral supplementation (83%), massage (80%), acupuncture (79%), chiropractic (77%), yoga (74%), and herbal medicine (72%). They were less familiar with meditation (69%), tai chi (61%), homeopathy (55%), hypnosis/Guided Imagery (GI) (52%), and Traditional Chinese Medicine (TCM) (49%). ID physicians were least familiar with qi gong (17%), Ayurveda (26%) and Healing Touch (HT)/Reiki/Therapeutic Touch (TT) (39%). ID physicians most recommended the following IM modalities: vitamins and mineral supplementation (80%, n= 252), massage (62%, n=247), yoga (52%, n=227), acupuncture (46%, n=241) and yoga (45%, n=214). ID physicians less frequently recommended chiropractic (33%, n=233), herbal medicine (32%, n=219), hypnosis/GI (28%, n=163) and tai chi (26%, n=189). Finally, they least recommended qi gong (6%, n=77), homeopathy (8%, n=173), TCM (11%, 158), Ayurveda (12%, n=95) and HT/Reiki/TT (16%, n=126).

## Conclusion

ID physicians are familiar with and recommend IM modalities, particularly BBM and MBBM. They are least familiar with and also least likely to recommend EBM and WMS based modalities.

